# Treatment of progressive multiple sclerosis with high-dose all*-trans* retinoic acid – no clear evidence of positive disease modifying effects

**DOI:** 10.1186/s42466-021-00121-4

**Published:** 2021-05-10

**Authors:** Christoph Ruschil, Evelyn Dubois, Maria-Ioanna Stefanou, Markus Christian Kowarik, Ulf Ziemann, Marcus Schittenhelm, Markus Krumbholz, Felix Bischof

**Affiliations:** 1 Department of Neurology & Stroke, Eberhard-Karls University, Tübingen, Germany; 2 Hertie Institute for Clinical Brain Research, Eberhard-Karls University, Tübingen, Germany; 3 Department of Hematology, Oncology, Clinical Immunology and Rheumatology, Eberhard-Karls University, Tübingen, Germany; 4 Department of Oncology/Hematology, Kantonsspital St Gallen, St Gallen, Switzerland; 5 Nervenärztliche Gemeinschaftspraxis, Konrad-Zuse-Str. 14, Böblingen, Germany

**Keywords:** Progressive multiple sclerosis, All-*trans* retinoic acid, Vitamin A, Lymphocyte subsets

## Abstract

**Background:**

All-*trans* retinoic acid (ATRA) is an acid derivative of vitamin A which is discussed as a promising candidate to ameliorate the disease course of multiple sclerosis (MS) by immunomodulation or even by promoting regeneration in progressive MS. Here we report a patient who significantly improved for MS related disability following administration of chemotherapy including ATRA for mitoxantrone-related acute promyelocytic leukemia and assess the effect of high-dose ATRA in three additional patients with progressive MS.

**Methods:**

Patients with progressive MS who had failed previous therapies were treated with high-dose ATRA. Patients underwent clinical and routine laboratory monitoring. Additionally, PBMCs were analyzed by flow cytometry for lymphocyte subsets.

**Results:**

ATRA was well tolerated and no pathological laboratory abnormalities were observed. After initial mild (not statistically significant) improvement of EDSS and mean MSFC z-score, ongoing disease progression was observed. One patient subacutely experienced severe cognitive and motor worsening. Cerebral MRI revealed persistent gadolinium-enhancing lesions. Flow cytometric alterations of peripheral blood naïve, central memory and effector memory CD4 and CD8 T cells, B lymphocytes, plasma cells, memory B cells, plasmablasts and natural killer (NK) cells did not reach statistical significance.

**Conclusions:**

Stand-alone therapy with ATRA did not ameliorate progressive MS in our limited cohort and we did not observe consistent alterations of T and B cell subsets. Intriguingly, application of ATRA may have caused marked disease exacerbation in one patient.

**Supplementary Information:**

The online version contains supplementary material available at 10.1186/s42466-021-00121-4.

## Introduction

While treatment options for patients with relapsing-remitting multiple sclerosis (RRMS) have improved tremendously during the last years, evidence of effective therapies for progressive disease is still scarce. Mitoxantrone reduces disease progression in secondary progressive multiple sclerosis (SPMS), but its use is limited by side effects, including the high risk for cardiotoxicity [1] and occasional therapy-related acute leukemia. In particular, therapy-related acute promyeloic leukemia (tAPL) has been linked to the inhibition of topoisomerase II with subsequent DNA double strand breaks [2]. Recently, ocrelizumab has been approved as the first drug to modestly delay disease progression in primary progressive multiple sclerosis (PPMS), while siponimod may delay disease progression in SPMS with superimposed relapses [3, 4]. Further compounds are still in the experimental phase or clinical testing, and hitherto unavailable.

All-*trans* retinoic acid (ATRA) is an acid derivative of vitamin A (retinol), which regulates a broad range of biological processes. ATRA is an essential part of the AIDA (all-*trans* retinoic acid and idarubicin) protocol [5] for treatment of tAPL, consisting of an anthracycline (idarubicin) chemotherapy in addition to simultaneous ATRA for remission induction, followed by 3 cycles of consolidation and maintenance therapy. ATRA seems to exert positive effects also in autoimmune diseases, including MS. In RRMS, results from a previous study showed improvement of MSFC but not EDSS in RRMS patients treated with vitamin A (initially with 25.000 IU/d for 6 months, followed by 10.000 IU/d foranother 6 months) [6]. An earlier study had already pointed towards beneficial effects of ATRA in combination with interferon-beta treatment by promoting suppressor cell function [7]. Moreover, in an animal model of MS, ATRA appeared to exhibit synergistic effects with atorvastatin and led to reduced secretion of the pro-inflammatory cytokine interleukin-17 and increased production of the anti-inflammatory interleukin-10 [8]. These findings are in line with earlier observations showing that oral Vitamin A ameliorates the course of experimental autoimmune encephalomyelitis (EAE) [9–11].

Via retinoic acid receptors (RARs) [12], ATRA has been suggested to induce a shift in T cell subsets from a Th1 to a Th2 type profile [11]. Moreover, proliferation and differentiation of Th17 cells [13, 14] is decreased by ATRA, e. g., by modulation of chemokines and chemokine receptors, including IL-1 receptor (IL-1R) up-regulation, IL-6R down-regulation [15] and an increase in IL-2 secretion, which is at least partly mediated by RARs [16]. In addition, alteration of other components of the immune system, including B cells and immunoglobulin production has been observed [17, 18]. Beside these immunomodulatory effects, there is evidencefrom animal models that ATRA might generate neurite outgrowth [19, 20], probably by interaction via nerve growth factor [21]. Taking these findings together, ATRA was considered a candidate for immunomodulation and regeneration in MS.

Here, we report an index patient with progressive MS, who improved clinically after therapy with ATRA plus an anthracycline-based chemotherapy regimen (AIDA protocol) for the treatment of tAPL, as well as the clinical outcome and flow cytometry results of three subsequent patients with progressive MS who received off-label high-dose ATRA treatment.

## Methods

### Standard protocol approvals, registrations, and patient consents

All patients were treated at the Center of Neurology, University of Tübingen, Germany (index patient 2002–2020, other patients 2015–2019). All patients consented to off-label treatment with ATRA after previous treatment had failed and provided written informed consent for the provision of blood samples for research purposes. The protocol for processing and analyzing peripheral blood samples was approved by the Ethics Committee of the University of Tübingen, Germany (029/2014BO2). Ethics approval of individualized treatment with ATRA with a defined protocol (including ≤4 treated patients) was not necessary under German ethics legislation (“Individueller Heilversuch”). The reasons for implementing the off-label use of ATRA for progressive MS were: (i) continued clinically relevant disease progression and failure of previous treatments with no alternative treatment options currently available, (ii) limited evidence of ATRA as a promising drug for autoimmune diseases from the index patient and literature as described above, (iii) good evidence from the AIDA protocol in support of good tolerability and safety profile of ATRA.

For control purpose, we used an internal cohort of progressive MS patients (*n* = 52) who presented in our outpatient clinic between 2014 and 2019 (retrospective analysis, control cohort from ethics approval 329/2019B01), further details are described in Supplementary S4.

### Patient characteristics and plan of treatment

All 3 patients had been diagnosed with progressive multiple sclerosis (2xPPMS, 1xSPMS) and fulfilled the McDonald criteria for MS [22] and Lublin revised criteria for progressive MS [23]. They had failed previous treatments and had a documented progressive course of disease, including progressive worsening of walking ability for at least 2 years before initiation of treatment with ATRA. None of the patients had any signs of acute relapse or acute exacerbation within the previous year (Table 1, patient characteristics and diagnostic data). They did not show significant alterations compared with retrospectively analyzed control patients concerning EDSS at baseline, age, age at disease onset and disease duration.

**Table 1:** Clinical characteristics of patients

Patient No	0 (Index)	1	2	3	Control cohort
Sex	female	female	male	male	female *n* = 31, male *n* = 19
Age	56	59	60	39	Mean = 53,9 ± 3.1 [range 28–82]
Course of MS	SPMS	PPMS	PPMS	SPMS	PPMS *n* = 26 SPMS *n* = 24
Age of disease onset	15 (RRMS) / 23 (SPMS)	52	58	29 (RRMS) / 35 (SPMS)	Mean = 40.6 ± 3.8 [range 18–71]
EDSS baseline	6.5	5.0	5.5	4.0	4.92 ± 0.23 [range 1.5–7.5]
Previous treatment	Azathioprine, betaferon, e MP q3m, mitoxantrone	Mitoxantrone, MP q3m	MP q3m	Glatiramer acetat, fingolimod, teriflunomide, MP q3m	various
Concomitant treatment	Botolinum toxin q3m	Fampridin, L-Thyroxin	Pramipexol	THC/CBD-spray	various

*PPMS* primary progressive multiple sclerosis, *SPMS* secondary progressive multiple sclerosis, *EDSS* extended disease severity score, *MP q3m* high dose intra-venous methyprednisolone (1000 mg/day for 3–5 days) every 3 months

**Fig. 1:**
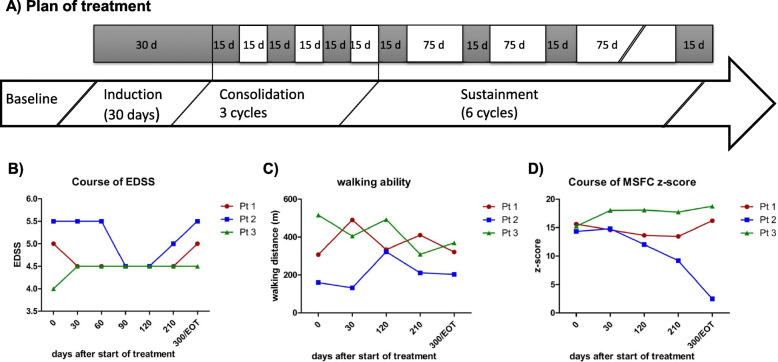
Schematic overview of treatment plan and clinical outcome. **a** Schematic overview of the planned treatment, consisting of 30 days of induction therapy followed by 3 cycles of consolidation therapy and 6 cycles of sustainment therapy. Due to lack of evidence and potential aggravation of disease activity, treatment was stopped after the 2nd cycle of sustainment / day 300. **b** Clinical course of EDSS and **c** clinical course of walking ability. Slight non-significant improvement was observed on day 210 that was not statistically significant and could not be proven on day 300. **d** MSFC z score did not show significant changes. All tests showed no significance (*p* < 0,05) in Friedman-test with Dunn’s post-hoc test

Similar to the ATRA administration in the AIDA-protocol, a 30-day induction phase with daily oral administration of ATRA (45 mg/m^2)^ was followed by 3 cycles of consolidation (daily medication during the first 15 days of a 30-day cycle) and multiple cycles of sustainment (3-month cycles with treatment during the first 15 days, planned for 6 cycles or until sustained disease progression) (Fig. 1a). One patient (patient 1) received 4 cycles of sustainment, patient 2 and 3 discontinued treatment after the 2nd cycle of sustainment (= day 300) due to disease progression. During treatment with ATRA, symptomatic therapies (e. g. anti-spastic) were continued, but no additional immunomodulatory drugs (except for intravenous corticosteroids in patient 2 for acute disease exacerbation) were administered.

### Clinical assessment

For maximal patient safety during off-label treatment, patients were assessed regularly (monthly during induction/consolidation phase and every 3 months at the beginning of each cycle of the sustainment phase). Routine laboratory investigations (including blood count, serum electrolyte, liver enzymes, infection parameters) were drawn at each visit and adverse events were documented. Clinical course was documented by EDSS (not blinded, performed by certified EDSS raters), measured maximal walking distance, and multiple sclerosis functional composite (MSFC) test, consisting of 9-hole-peg-test (9HPT), 25-ft walking time (25FWT) and paced 3″ auditory serial addition test (PASAT 3″).

### Flow cytometry staining

Peripheral blood mononuclear cells (PBMCs) were isolated from EDTA blood using a Ficoll gradient and frozen (− 80 °C) until further analysis. For flow cytometry, the following antibodies were used: anti-human CD3-APC (1/100) clone UCHT1, anti-human CD4-FITC (1/100) clone SK3, anti-human CD8-PerCP (1/100) clone SK1, anti-human CD19-FITC (1/100) clone HIB19, anti-human CD27-PE (1/50) clone L128, anti-human CD38-APC (1/50) clone HIT2, anti-human CD56-PE (1/50) clone B159, anti-human CD197(CCR7)-PE (1/50) clone 3D12 (all BD Biosciences), anti-human CD45RA-APC (1/50) clone HI100 (BioLegend). As a reference, analyses were additionally performed in 10 healthy controls. Gating strategy for CD4/CD8-T cell subsets, NK cells and B cell-subsets is displayed in Supplementary Fig. S1.

### Statistical analysis

For graphical and statistical representation, GraphPadPrism, Version 5.0 and customized R scripts [24] were used. For comparison of clinical parameters and lymphocyte subpopulations at different timepoints, the non-parametric Friedman-test with Dunn’s post-hoc analysis was applied, *p*-values were adjusted for multiple testing using the Bonferroni correction. Wilcoxon rank sum test was applied for comparisons between ATRA treated patients and the control cohort. Adjusted values for *p* < 0.05 were considered statistically significant. All values are displayed as mean ± sd [95% confidence interval (CI) lower; 95% CI upper] unless stated differently.

## Results

### Index patient

Our hypothesis that ATRA may exert positive effects on the course of progressive multiple sclerosis emerged from the clinical observation of a woman with SPMS who improved significantly in walking ability and EDSS after receiving the AIDA protocol for the treatment of tAPL, which includes high doses of ATRA.

RRMS was first diagnosed in 1978, when she presented with hemianopsia that responded well to glucocorticoid treatment. CSF analysis revealed pleocytosis and positive oligoclonal bands (OCB) and evoked potentials were prolonged. MRI showed spinal, infratentorial, juxtacortical and periventricular lesions, typical for MS. In the following years, she experienced two more clinical relapses, but from 1986 onwards a secondary progression with reduction of her walking ability and left dominant tetraparesis was documented. The patient had been initially treated with azathioprine (1978–1980), followed by glucocorticoids (in the first years irregularly after relapses, in the next several years 3-4x/year as treatment for progressive disease) until 1998. Despite treatment, the patient essentially lost her walking ability (documented EDSS 6.5) in 1999. Subsequently, mitoxantrone was administered from 2000 to 2002 (initial dose 12 mg/m^2^ i.v./every 3 months, cumulative dose 72 mg/m^2^) until acute promyeloic leukemia was diagnosed in 04/2002. The length of exposure to mitoxantrone was compatible with the diagnosis of mitoxantrone therapy-related leukemia (tAPL). Following the AIDA protocol for tAPL, induction therapy with Idarubicin (12 mg/m^2^ on day 2, 4, 6, 8) and cytarabin (Ara-C 100 mg/m^2^/24h on day 1) accompanied by ATRA (45 mg/m^2^ until complete remission) was initiated. Three cycles of consolidation (anthracycline-based and cytarabine, each combined with ATRA for 15 days in 30-day-cycles) were administered subsequently, followed by 6 cycles of maintenance therapy (6-mercaptopurine daily, methotrexate weekly plus ATRA during the first 15 days of 3-month-cycles). Treatment was successful, with a stable complete molecular remission ever since (up to date 2020).

Although no further immunomodulatory/immunosuppressive therapy was administered since 2004, MS symptoms continuously improved after the end of the tAPL therapy. The patient regained her walking ability and was later able to walk 120 m with bilateral assistance. Motor function of the arms increased and the EDSS improved slightly to 6.0. The patient underwent botulinum toxin injections (facial spasms) since 2004 and regular physiotherapy without further symptomatic drug therapies.

Based on this favorable outcome of the index patient and given the above-mentioned research data on immunomodulatory effects of ATRA, we hypothesized that administration of high-dose ATRA as in the tAPL protocol may have positively modified the course of disease of progressive multiple sclerosis.

### ATRA is well tolerated and causes no abnormalities in routine laboratory parameters

All three subsequent patients complained about light headache during the first days of each cycle. Two out of three patients had mild flu-like symptoms after the first 30 days of induction, which resolved without treatment. One patient complained about mild worsening of a psoriatic exanthema and reported mild flushes. No serious treatment-related adverse events were observed during treatment with ATRA. Routine safety testing showed no relevant changes in WBC, serum electrolytes, infection parameters, and liver or kidney function.

### No changes in clinical outcome

Clinical outcome was assessed by EDSS rating, testing of walking ability and MSFC. For two out of three patients (not including the previous index case), initial improvement in EDSS after 120 days was observed (Fig. 1b) (Baseline: 4.83 ± 0.44 [CI 3.99; 5.69]; d120 4.5 ± 0 [CI not applicable]). However, this (non-significant) improvement could not be sustained. Instead, asubsequent clinical worsening was observed and at day 300 (after 2 cycles of sustainment therapy) EDSS returned to baseline values (d300: 5.0 ± 0.29 [CI 4,43; 5.56]). Compared with the control cohort (baseline EDSS 4.92 ± 0.23 [CI 4.49; 5.34]), the annual worsening (ΔEDSS) of 0.17 ± 0.1 [CI -0.02; 0.36] was within the expected range without significant improvement after treatment (control ΔEDSS 0.23 ± 0.09 [CI 0.05; 0.40],: *p* = 0.983; effect size W = 74.5).

Maximal walking ability (Fig. 1c) at baseline was 328 m ± 103 m [CI 209.9, 444.1] and initially improved at day 120 to 382 m ± 55 m [CI 235.7, 529.6] (mean delta change 71 m ± 72 m). Nonetheless, this change was not statistically significant. Yet, at the end of treatment/day 300, walking ability reached again a level below baseline with maximal walking ability of 298 ± 49 m [CI 212.2, 383.2]. Thus, no overall clinical effects on walking ability could be observed.

MSFC Test (containing 25FWT, 9HPT, 3”PASAT, Fig. 1d) showed similar tendencies with minimal, not statistically significant or clinically relevant improvement at day 30 and day 120 that were not sustained at day 210 and day 300 (baseline z-score: 15.1 ± 0.38 [ci 14.3; 15,8], d300: 12.5 ± 5.05 [ci 2.6; 22.4]). Analysis of the single components (see Supplementary Fig. S2) showed improvement of 9HPT at days 30, 120 and 300 against baseline (moderate effect size, Kendall W = 0.424), most likely due to training effects. For 25 ft walk test or 3”PASAT no significant changes were observed.

### Lymphocyte subsets

**Fig. 2:**
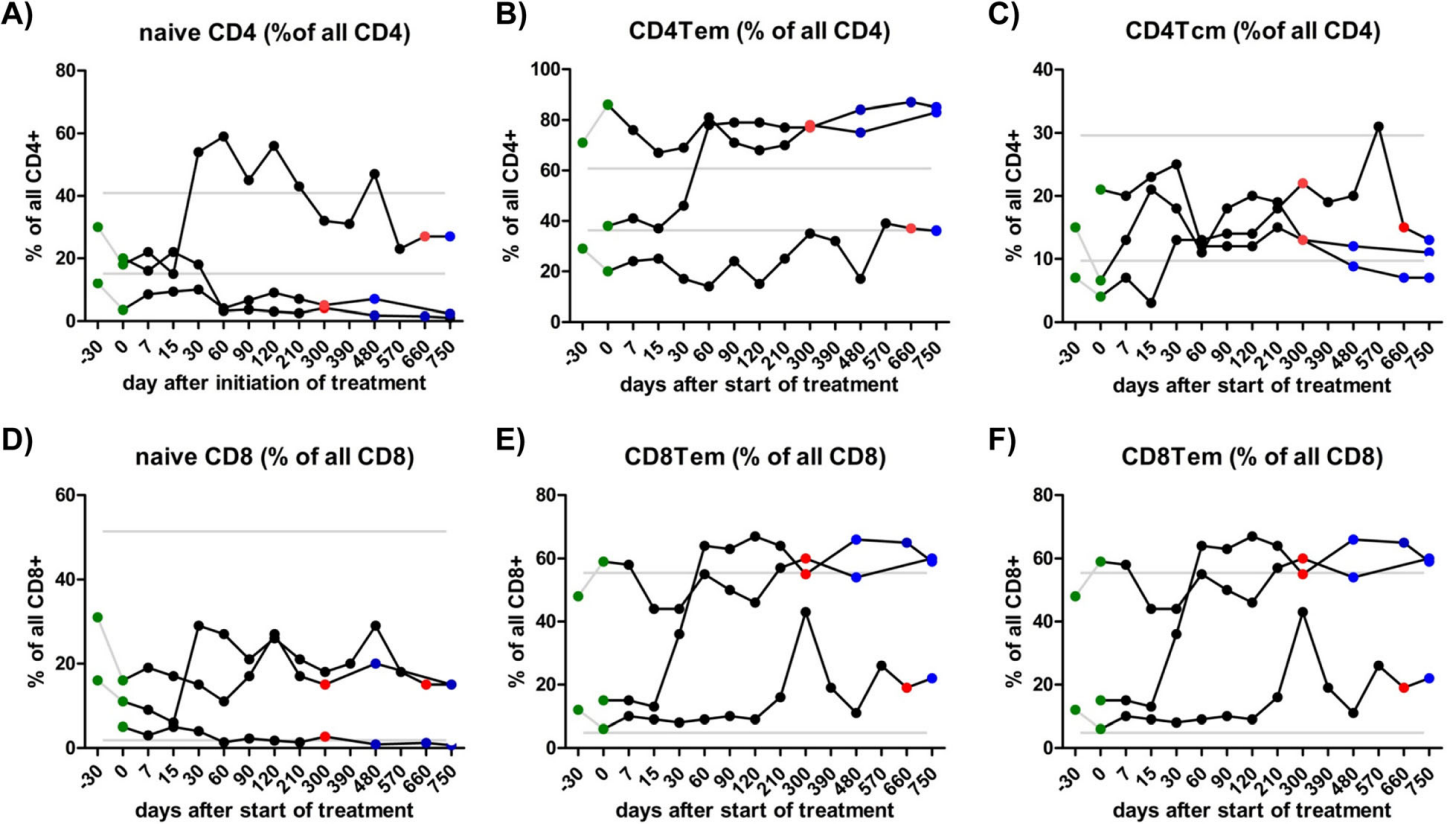
High heterogeneity in CD4 / CD8 T cell subsets with no obvious alteration by ATRA. Central memory CD4+ T cells (CD4+ Tcm), naïve CD4+ T cells and effector memory CD4+ T cells (CD4+ Tem) as well as central memory CD8 T cells (CD8+ Tcm), naive CD8+ T cells and effector memory CD8+ T cells (CD8+ Tem) were tested before (green dots), during (black dots) and in the follow-up period after (blue dots) treatment with ATRA, red dots indicate end of treatment. High heterogeneity was observed with no evidence of direct alteration by induction of treatment. Grey lines indicate range of normal values (mean +/− 2*SD) obtained as reference from 10 healthy controls. All tests showed no significance (*p* < 0,05) in Friedman-test with Dunn’s post-hoc test

At baseline and during treatment with ATRA, peripheral blood samples were obtained from all patients and cells were analyzed by flow cytometry. After induction therapy with ATRA, we observed a proportional increase of naive CD4+ and naive CD8 T+ cells in one patient (patient 1), whereas another patient (patient 2) presented a proportional increase of CD4+ and CD8+ effector memory T cells (Fig. 2). However, compared to normal population or healthy controls (i.e., not treated with ATRA), we did not observe any significant differences.

**Fig. 3:**
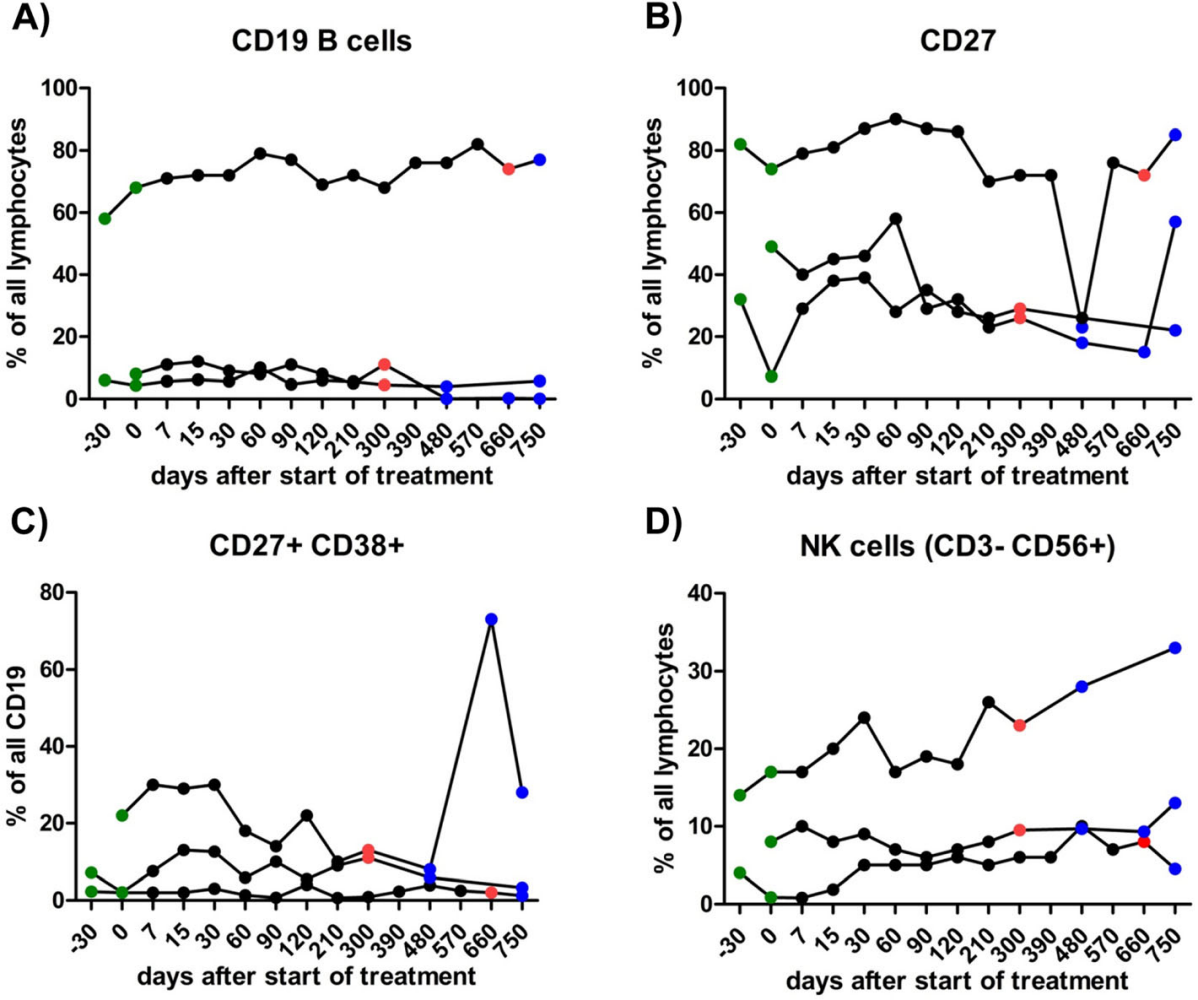
Frequency of B lymphocytes, plasma cells and NK cells. Frequency of CD19+ B lymphocytes, CD19 + CD38+ plasma cells, CD19 + CD27+ memory B cells and CD56+ NK cells did not significantly differ during treatment of ATRA (Friedman test with Dunn’s post-hoc test *p*<0.05)

The frequencies of central memory CD4+ or CD8+ T cells (Fig. 2), CD19+ B lymphocytes, CD19 + CD38+ plasma cells, CD19 + CD27+ memory B cells and CD56+ NK cells (Fig. 3) were not affected by ATRA.

### Disease exacerbation in one patient

**Fig. 4:**
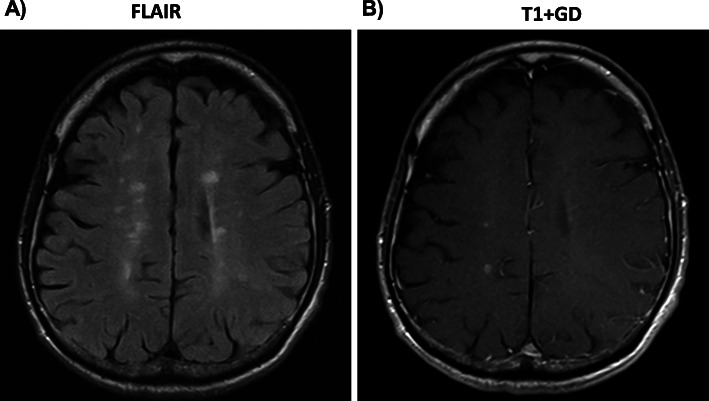
MRI imaging of patient 2 displaying disease activity. **a** FLAIR and **b** T1 weighted MRI with application of gadolinium at day 300 after start of ATRA revealed multiple gadolinium-enhancing lesions

One patient (patient 2) experienced severe cognitive and motor worsening after the first cycle of sustainment therapy (day 210). Cerebral MRI revealed gadolinium enhancing lesions. The patient received high-dose glucocorticoid treatment and the 2nd cycle of sustainment therapy with ATRA was continued. However, symptoms progressed. Repeated MRI 3 months later, showed persisting and new gadolinium enhancing lesions (Fig. 4). ATRA therapy was halted and the patient received another cycle of high-dose glucocorticoid treatment followed by rituximab (1 g every 6 months), which stabilized the disease course. No new T2 or persistent Gd + MRI lesions were observed in the following 2 years. This disease exacerbation was unexpected as none of the 50 patients in the control cohort showed similar disease activity on repeated MRI scans.

### Clinical outcome

While a slight, non-significant clinical improvement was noted at initial stages during induction therapy, all patients experienced clinical worsening in subsequent cycles, with an overall reduced walking ability noted at day 300. Therefore, treatment with ATRA was discontinued in all patients. MRI (FLAIR, Gd-enhanced T1) of patient 1 and 3 remained stable during and after treatment.

## Discussion

All-trans retinoic acid (ATRA) is known to interact via multiple ways with the immune system and previous studies have suggested that ATRA could shift the imbalance in multiple sclerosis from a Th1/Th17 to Th2/Treg T cell phenotype [25], potentially facilitating clinical improvement in MS or even inducing neuroregeneration. Here we report a single index patient who improved significantly after treatment with a high-dose ATRA-containing regimen (AIDA protocol for tAPL). This observation is in line with reports of beneficial effects after ATRA treatment in other autoimmune disorders, including inflammatory bowel disease [26] and systemic lupus erythematosus [27], where ATRA has been shown to reduce proteinuria in lupus nephritis. The positive effects noted in our index patient are also in line with evidence of efficacy of ATRA in dampening inflammatory processes in animal models. In particular, ATRA has been associated with attenuation of disease activity in EAE models [9, 10] along with attenuation of neuroinflammation following global ischemia and rescue of neurons in a gerbil model [28].

Despite the suggested clinical improvement associated with ATRA therapy in the index patient, we could not observe any significant amelioration of clinical symptoms in three further patients treated with high-dose stand-alone ATRA. In this small cohort, we were not able to demonstrate any consistent shift in T and B cell subsets. Yet, as the flow cytometry results exhibited high variability already at baseline, treatment induced changes may have remained undetected. Some further methodological limitations should be considered for an accurate interpretation of our results:a) The population treated with ATRA was small, heterogenous and had failed previous treatment attempts. The small population size per se may account for type II errors. However, in this small cohort, no striking ATRA effects in these exemplary MS patients were noted. The small cohort was heterogenous and included patients with primary and secondary progressive MS. Although genetic and histopathological features as well as the natural course of disease indicate a common “progressive stage” of MS [23, 29], it cannot be ruled out that PPMS and SPMS are two different entities. Moreover, it has been suggested to categorize MS in three different stages: early/initial stage driven by systemic inflammation, a second stage of compartmentalized inflammation in the CNS and a third stage with prominent neurodegeneration [30]. Taking the promising experience from the index patient, we hypothesized that ATRA might hold potential for attenuating neurodegeneration. With a range of disease duration between 2 and 10 years, our patients were most likely in the second to third stage of MS. However, the patient with the shortest disease duration (2 years) experienced disease exacerbation with new Gd-enhanced lesions on MRI. Thus, we cannot exclude that differential ATRA effects could be expected in patients with different MS types or at different MS stages. Also, regarding the previous failed treatment attempts, we consider it unlikely that relevant carry-over effects persisted when treatment with ATRA was initiated. More importantly, the repeated failures of treatment attempts and the severe disability (EDSS 4.0–6.0) of our patients indicate a severe MS course. Thus, we cannot exclude that a “floor-effect” may have confounded our results. As seen in previous studies, rigorous selection of inclusion criteria, including young age, short disease duration and MRI activity might unmask treatment effects. Nonetheless, we considered ATRA as off-label treatment to be ethically justified only in selected patients with aggressive disease course, persistent clinical worsening and after all other available treatment options had been exhausted, even at the cost of introducing bias in patient selection.

b) While the index patient had received ATRA and additional idarubicin during induction, cytarabin during consolidation and 6-mercaptopurin during maintenance stages of the AIDA protocol, the prospectively treated three patients received only ATRA monotherapy. Idarubicin has not been previously studied in multiple sclerosis. Cytarabin is part of high-dose immunosuppressive treatment in combination with autologous hematopoietic stem cell transplantation in multiple sclerosis [31], but monotherapy has failed to improve neurological outcome [32]. Certainly, 6-mercaptopurin (or its prodrug azathioprine) has well proven effects on the course of disease in multiple sclerosis [33] but it seems very unlikely that a limited treatment period during 6 cycles of maintenance could have induced the observed amelioration of the index patient.

c) Effects like neuronal outgrowth or neuroregeneration promoted by ATRA evolve slowly and the duration of treatment or the subsequent follow-up might not have been sufficient to detect slight but steady improvements as the ones observed in the index patient for nearly 20 years. Therefore, the observational period may have been too short. However, the clinical worsening and acute exacerbation in at least one patient prompted us to withdraw ATRA treatment.

On the other hand, we contemplated whether ATRA might induce detrimental effects in MS. Patient 2 in our cohort experienced disease exacerbation with clinical worsening and acute gadolinium enhancing lesions during the treatment period. Although this finding was unexpected and a causative role of ATRA cannot be formally proven, detrimental effects of ATRA in experimental settings have been previously reported. In a spinal cord injury model, application of ATRA increased the Treg population and reduced activation of Teff cells, which was paradoxically associated with decreased neuronal survival [34]. Additionally, in an experimental mouse model of lupus-like disease, ATRA promoted neuroinflammation with clinical disease exacerbation and increased circulating plasma cells, autoantibodies and total IgG [35, 36]. A potential explanation for this paradoxical cerebral inflammatory process might be that in vivo, retinoic acid interacts via multiple pathways with immunological cascades, besides the Th1/Th2-pathways. For example, retinoic acid has been shown to increase B cell proliferation and plasmocytic cell differentiation [37]. Considering the crucial involvement of antibody and B cell mediated-processes especially at early disease stages of MS [38], an ATRA-mediated propagation of B cell proliferation may explain clinical worsening. Of note, patient 2, who experienced relapse symptoms, displayed higher relative plasmablasts (percentage of total B cells) than the other subjects.

## Conclusions

ATRA may promote or inhibit inflammation dependend on the stage of disease [39] or the overall susceptibility of the inflammatory environment. In combination with other immunomodulatory substances such as atorvastatin [8], interferone-beta [40] or Vitamin D [41], − or even in combination with other immunusuppressive drugs like cytarabin or 6-mercaptopurin - high-dose ATRA might indeed regulate inflammatory processes. Eventually, this could be the case with our index patient, who received ATRA not as stand-alone therapy but in combination with chemotherapy. As a monotherapy, at least in our limited cohort, and possibly depending on the stage of disease, high-dose ATRA failed to promote clinical improvement.

## Supplementary Information

**Additional file 1: Supplementary Figure S1.** Schematic overview for flow cytometry. **Supplementary Figure S2.** Subanalysis of single components of the MSFC test. **Supplementary Figure S3.** Additional analysis of flow cytometry results. **Supplementary S4.** Control cohort for progressive multiple sclerosis.

## Data Availability

Not applicable.

## Abbreviations

AIDA: All-*trans* retinoic acid and idarubicin (protocol)
Ara-C: Cytarabine
ATRA: All-trans retinoic acid
CSF: Cerebro-spinal fluid
EDSS: Expanded disability status scale
Gd: Gadolinium
IL-1R: Interleukin-1 receptor
MRI: Magnetic resonance imaging
MS: Multiple sclerosis
MSFC: Multiple sclerosis functional compound
NK cells: Natural killer cells
OCB: Oligoclonal bands
PMBC: Peripheral blood mononuclear cells
PASAT 3″: Paced 3″ auditory serial addition test
PPMS: Primary-progressive multiple sclerosis
RARs: Retinoic acid receptors
RRMS: Relapsing-remitting multiple sclerosis
SPMS: Secondary-progressive multiple sclerosis
tAPL: Therapy-related acute promyeloic leukemia
25FWT: 25-ft walking time
9HPT: 9-hole-peg-test
