# Supplementary material for: Treatment of progressive multiple sclerosis with high-dose all-trans retinoic acid – no clear evidence of positive disease modifying effects
Source: Neurol Res Pract. 2021 May 10;3:25. doi: 10.1186/s42466-021-00121-4 (PMC8108354; doi:10.1186/s42466-021-00121-4)
Supplement: Supplementary file 1 — Additional file 1: Supplementary Figure S1. Schematic overview for flow cytometry. Supplementary Figure S2. Subanalysis of single components of the MSFC test. Supplementary Figure S3. Additional analysis of flow cytometry results. Supplementary S4. Control cohort for progressive multiple sclerosis. [file 42466_2021_121_MOESM1_ESM.docx]

**Supplementary Figure S1**


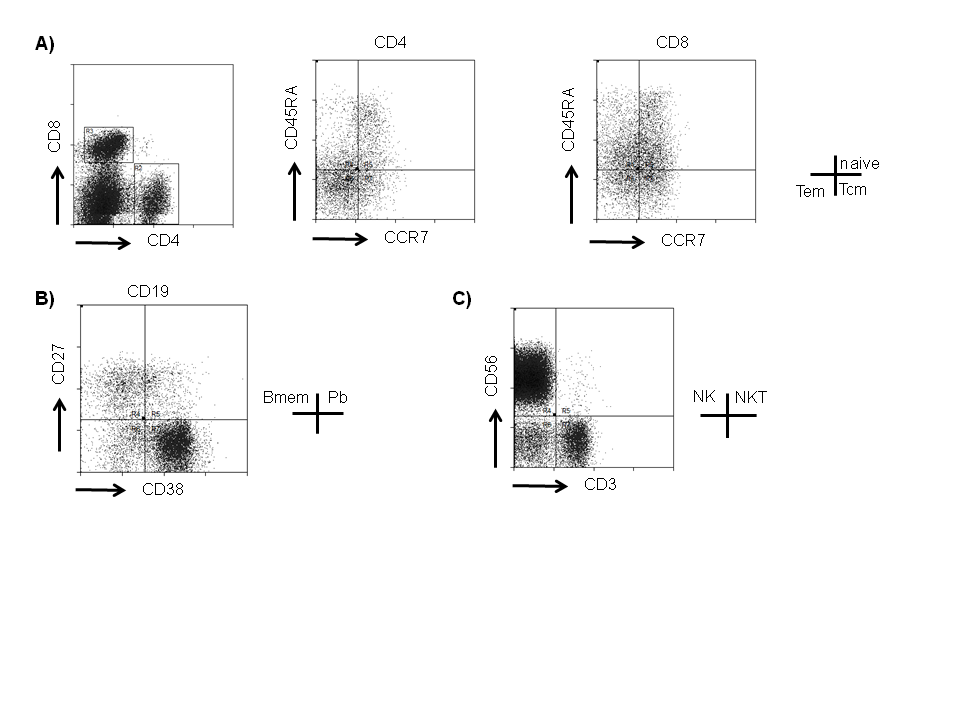


**Supplementary Figure S1: Schematic overview for flow cytometry**

A) Expression of CCR7 and CD45RA was determined on live CD4 and CD8 cells as displayed, ; B) expression of CD38 and CD27 was determined on live CD19 to distinguish Bmem and Pb; C) expression of CD56 and CD3 was applied for natural killer cells and natural killer T cells.

Tem: T effector memory (CD45RA- CCR7-), Tcm T central memory (CD45RA- CCR7+); Bmem = B cell memory cells(CD27+ CD38-); Pb = Plasmablasts (CD27+CD38+); NK = natural killer cells (CD56+CD3-); NKT = natural killer T cells (CD3+CD56+)

**Supplementary Figure S2:**


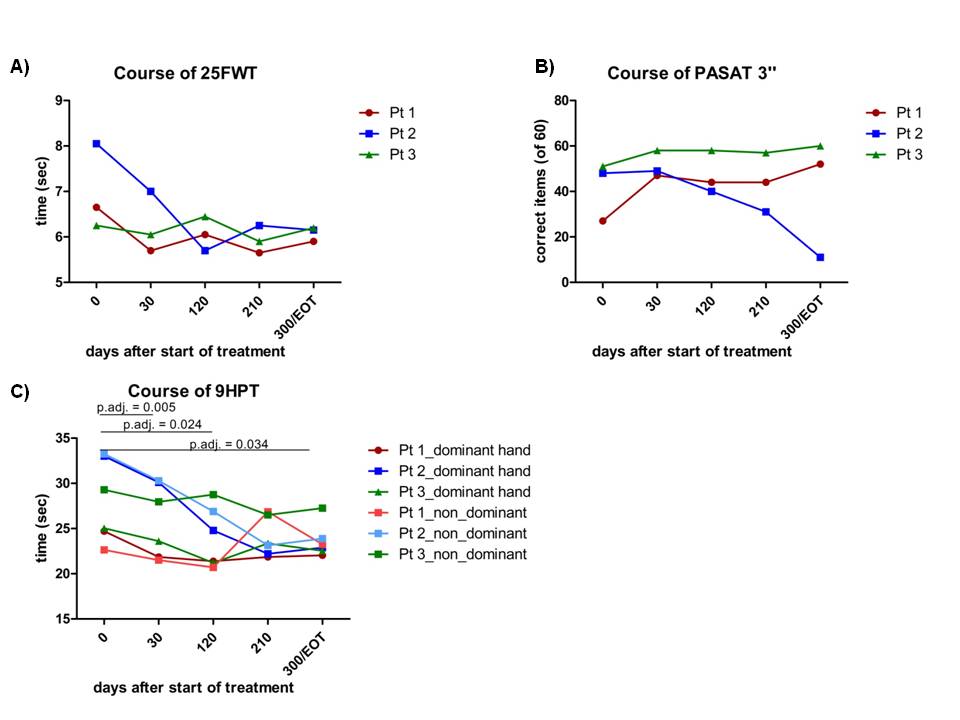


**Supplementary Figure S2: Subanalysis of single components of the MSFC test**

A) 25 foot walk test, B) PASAT3'' and C) 9 hole peg test’’. No significant changes in 3''PASAT or 25 foot walk test were observed. Improvement of 9HPT against baseline on d30, d120 and d300/EOT, most likely due to training effects, were observed (moderate effect size, Kendall W = 0.424). Friedman-test with Dunn’s post-hoc test and Bonferroni correction method for multiple testing, p-values as indicated in the graphs, otherwise not significant.

**Supplementary Figure S3:**


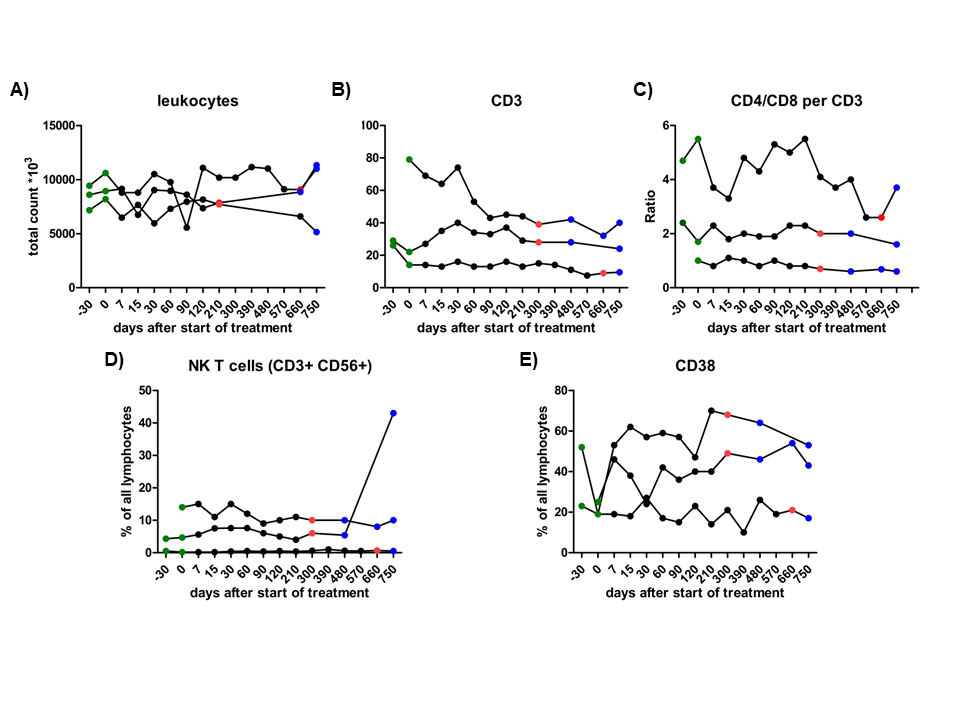


**Supplementary Figure S3:** **Additional analysis of flow cytometry results**

A) Leukocytes, B) CD3+T cells, C) ratio of CD3+CD4+/CD3+CD8+, D) NK T cells (CD3+CD56+) and total CD38+ were assessed before (green dots), during (black dots) and in the follow-up period after (blue dots) treatment with ATRA, red dots indicate end of treatment. High heterogeneity was observed with no evidence of direct alteration by induction of treatment.

**Supplementary S4 - Control cohort for progressive multiple sclerosis:**

We analyzed a retrospective control cohort of patients who had presented in our outpatients clinic between 2014 and 2019. Inclusion criteria were: diagnosis of primary or secondary progressive multiple sclerosis, was clinical follow-up > 1 year, and at least one MRI during that time (female n = 31, male n = 19, further characteristics, e.g. age and EDSS are displayed in table S4). From these 50 patients, 35 were untreated, the other received various treatments (rituximab n = 4, ocrelizumab n = 2, repeated high-dose i.v. methylprednisolon n = 4, dimethyl fumarate n = 1, interferone-beta n = 2; natalizumab n = 2). Mean EDSS at baseline was 4.92 ± 0.42 [CI 4.49; 5.34], after one year, mean EDSS was 5.15 ± 0.21 [CI 4.73; 5.57]; For 40 patients, follow-up data > 2 years could be analyzed, mean EDSS after 2 years was 5.55 ± 0.24 [CI 5.08; 6.01]. Therefore, the annual worsening of EDSS was 0.2­3 ± 0.09 [CI 0.05; 0.40] for the first year or a mean worsening per year of 0.225 ± 0.05 [0.11; 0.22] during the first 2 years. All patients had a recent MRI at baseline, 11/50 at this timepoint had worsening in comparison to reported older MRI (11 patients with new or enlarged T2 lesions, 1 small GD enhancing lesion). For 43/50 patients, at least one follow-up was available (mean time interval 22.5 ± 1.8 months [CI 18.8 - 26.2]; [range 4 - 51]). During this interval 6/43 patient had signs of disease progression in MRI (4 new T2 lesion, one single spinal Gd enhancing lesion, one single cerebral Gd enhancing lesion), the other MRI were reported as stable to prior MRI.

|  | N patients | Mean ± SEM | 95% CI | Range [min, max] |
| --- | --- | --- | --- | --- |
| Course of disease | PPMS = 26  SPMS = 24 | N/A | N/A | N/A |
| Gender | Female = 31  Male = 19 | N/A | N/A | N/A |
| Age | n = 50 | 53.92 ± 1.61 | CI 50.76; 57.07 | 26 – 77 |
| EDSS_baseline | n = 50 | 4.92 ± 0.42 | CI 4.49; 5.34 | 1.5 - 7.5 |
| EDSS after 1 year | n = 50 | 5.15 ± 0.21 | CI 4.73 - 5.57 | 2.0 - 7.5 |
| Mean annual delta 1 year | n = 50 | 0.23 ± 0.09 | CI 0.05; 0.40 | -1.0 - 2.5 |
| EDSS after 2 years | n = 40 | 5.55 ± 0.24 | CI 5.08; 6.01 | 2.0 - 8.0 |
| Mean annual delta 2years | n = 40 | 0.225 ± 0.05 | CI 0.11; 0.22 | -0.25 - 1.25 |
